# Surface Effect of Nano-Roughened Yttria-Doped Zirconia on Salivary Protein Adhesion

**DOI:** 10.3390/ma14216412

**Published:** 2021-10-26

**Authors:** Muhammad Naeem Iqbal, Zhijian James Shen, Tore Bengtsson, Mirva Eriksson

**Affiliations:** 1 Department of Materials and Environmental Chemistry, Stockholm University, 10691 Stockholm, Sweden; muhammad.iqbal@mmk.su.se (M.N.I.); zhijian.shen@mmk.su.se (Z.J.S.); 2 Department of Molecular Biosciences, The Wenner-Gren Institute, Stockholm University, 10691 Stockholm, Sweden; tore.bengtsson@su.se

**Keywords:** 3 mol% yttria partially stabilized zirconia ceramics (3Y-TZP), saliva, nano-surface roughness, protein adsorption, alpha-amylase adsorption

## Abstract

Biocompatibility of yttria (3 mol%) stabilized zirconia ceramics, 3Y-TZP, was affected to a large degree as a result of protein adsorption from human saliva that in turn depends on materials surface properties. Variable nano-roughness levels in 3Y-TZP discs were characterized and tested for specificity and selectivity with respect to size and uptake for human salivary protein.

## 1. Introduction

Zirconia (3Y-TZP) ceramics have the highest fracture toughness and strength in ceramics and thus 3Y-TZP has been increasingly applied in the field of dentistry. It has commonly been recognized as a bio-inert in the sense of its ability to promote the bio-interfacial integration with both soft and hard tissues [1]. Differences in the surface properties of restorative materials such as surface topography is known to be a dominant factor in the adsorption of proteins at the supra- and subgingival levels [2]. The surface of a material is the first site of interaction where biological molecules, such as proteins from saliva or plasma, reside on for a significant period, ultimately regulating the surface chemistry and further biofilm formation. It is well known that protein and antibodies from plasma and saliva selectively adsorb on different surfaces, even on materials of variable composition [3]. Likewise, *Candida* binding have shown variable binding to different 3Y-TZP surfaces, with and without saliva treatment [2,4]. In these observations, materials have shown subtle but significant differences in protein and bacterial adsorption. Available literature on material characterization of 3Y-TZP surface topography is at the sub-micron level with the lower threshold values between 0.2 and 0.3 µm. However, the interaction between protein from saliva and the 3Y-TZP surface occurs at nanoscale as the biomolecules in their monomeric or dimeric forms are a few nanometers in size (<100 nm) [5,6].Understanding biomolecule and material surface interactions at this scale is of critical importance in biomedical technologies, in particular for proper function and longevity.

A general approach is developed for understanding the bio-interfacial integrations of 3Y-TZP ceramic by manipulating the surface roughness at micron and nanoscale in an in vitro setting. Our own experimental and clinical observations have led us to hypothesize that the difference in surface structure of 3Y-TZP, mainly at nano level, shows variable response when exposed to human salivary proteins. We have therefore designed three different surface architectures and incubated them in saliva to study the protein uptake. Tailor made 3Y-TZP disc surfaces were as-sintered, polished, and sand blasted. Sandblasting is a clinically practiced surface roughening technique where abrasion with sharp airborne particles can potentially generate cracks a few micrometers deep together with some surface nano-roughening [7,8]. Studying the adsorption of salivary protein is important because it is the first step towards the formation of plaque and microbial adhesion [2]. We have chosen human salivary α-amylase for visualization of protein adsorption on different surfaces of 3Y-TZP discs using CSLM imaging. The choice of α-amylase for visualizing is critical with respect to the clinical role it plays for the maintenance of oral health [9,10,11]. Abundantly available salivary alpha-amylase binds with the early colonizing bacterium and hence initiates formation of dental plaque, which in turn initiates oral diseases, such as dental carries and periodontitis [12,13]. 

## 2. Materials and Methods

### 2.1. Preparation and Characterization

3Y-TZP discs were formed and sintered at 1450 °C for 1 h in air. Henceforth, samples were classified as three types, namely, “Sintered”, “Polished”, and “Sand Blasted”. “Sintered” discs were taken directly after sintering without applying any post-sintering treatment. “Polished” discs were polished using LaboForce-100, with gradually finer polishing media. “Sand blasted” discs were prepared at 4 bar pressure, with a distance of around 2 cm between disc and nozzle of alumina blasting media. Discs were characterized using scanning electron microscopy (SEM) and optical interferometry for surface architecture at nano and micron levels, respectively. Further detail is provided in the Supplementary Materials.

### 2.2. In Vitro Study of the Adsorption of Protein from Human Saliva

To simulate in vivo conditions, freshly collected human saliva from a healthy donor was used as a source of protein. Practically, 1 part of saliva was diluted in 46-part phosphate buffer saline (PBS) at pH 6.4 and used as a stock for incubating 3Y-TZP discs. Adsorption of the total amount of proteins from human saliva was measured using bicinchoninic acid (BCA) assay. Standard curves of saliva dilution were made beforehand at appropriate dilution level. In parallel, the actual amount of protein in saliva was determined using a known standard of pure human salivary α-amylase and bovine serum albumin (BSA), further experimental details in Figures S1–S5. Additionally, the immunolabelling technique was used to show the presence of adsorbed protein (α-amylase) from saliva on to 3Y-TZP discs. The general scheme of immunolabelling and microscope experiments is presented in Figure S1.

## 3. Results and Discussion

**Figure 1:**
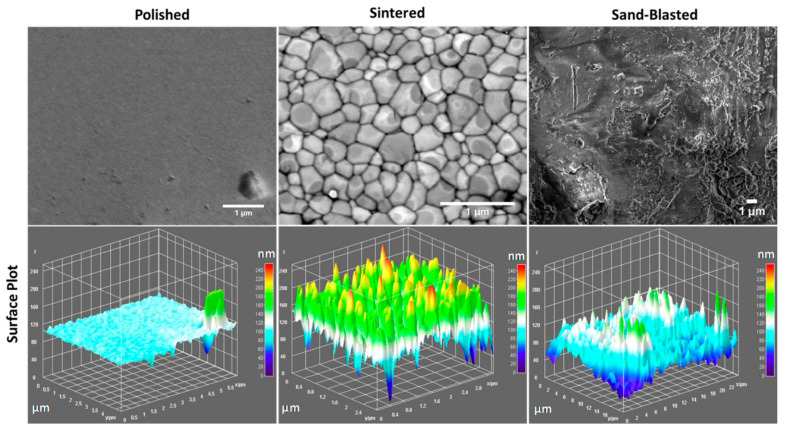
SEM images of the three types of surfaces of 3Y-TZP ceramics with their respective surface plots.

Sintered discs have a glossy looking surface, whereas the polished samples have a flattened mirror like surface. On the contrary, sand blasted 3Y-TZP disc surfaces turned from glossy to blurry and hazy. The SEM images taken on the surface of representative 3Y-TZP discs from each group are shown in Figure 1. Information on the surface structure of polished, sintered, and sand blasted discs were extracted from respective SEM images with the aid of Image J software (Figure 1). The existence of surface features such as faceted grains, pores, grain boundaries, and voids at the grain boundary junctions appears obvious in the sintered sample of 3Y-TZP. The size of individual grains is in the range of 50–500 nm, whereas that of voids at grain boundaries is around a few tens of nanometers. Such surface features are removed to a large degree by polishing. Sandblasted discs of 3Y-TZP on the other hand exhibit mainly micron sized roughened features, which can be clearly seen in the SEM images of Figure 1, with a small amount of nano roughness. However, comparison of surface plots of sintered and sand blasted discs showed removal of sharp peaks from the grains, resulting in the loss of fine surface structure. Blasting creates a new surface microstructure with micro cracks and chipping along the surface. At some points, these cracks can reach deeper into the surface whereby the impact can lead 3Y-TZP into local phase transformation, from tetragonal to monoclinic phase [14]. 

**Figure 2:**
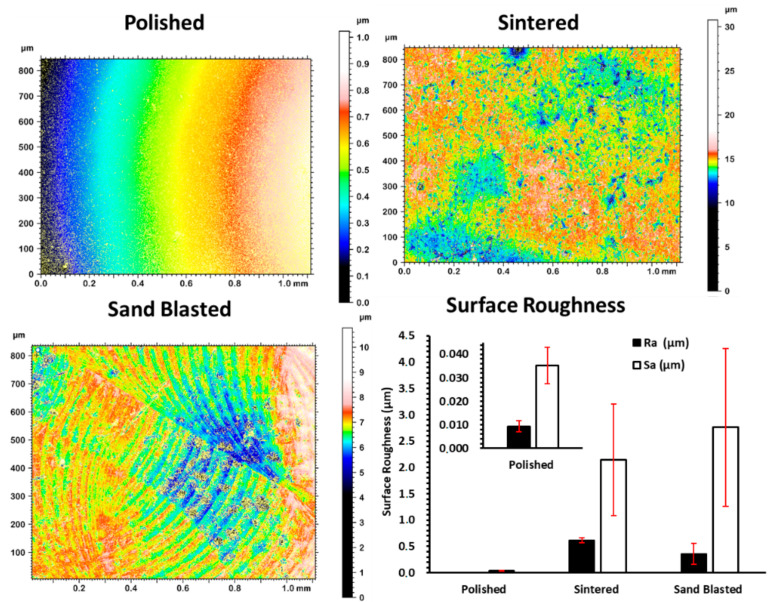
Surface topographic measurement of the as-sintered disc and after different surface treatments, generating polished and sand blasted surfaces and plotted surface roughness of each variant. Error bars are standard deviation.

The measured average surface roughness parameters Ra and Sa values for each of the discs are shown in the graph of surface roughness Figure 2. The average values of surface roughness parameters of polished discs were significantly lower than the values for sintered discs. However, paired *t*-test showed no significant difference between the sand blasted and sintered discs with *p*-values for Ra and Sa larger than 0.05. Sintered 3Y-TZP showed considerable homogeneous distribution of open porosity at the grain boundaries and sharp edges when compared to sand blasted specimens. Sand blasted samples had pronounced surface grooves, as presented in Figure 1 and Figure 2.

SEM images of a sintered sample (Figure 1) show individual grains having slight difference in contrast from light to dark grey due to their crystallographic orientation. Both the SEM and topographic measurement with profilometry show absence of close-up sharp profiles of individual grains for polished samples. Sand blasting treatment on the sintered discs showed a shift from nanoscale roughness profile to more micron level surface roughness, that is clearly visible in the surface plot of Figure 1 and deep surface scratches in Figure 2.

### 3.1. In Vitro BCA Assay for Total Protein Adsorption

**Figure 3:**
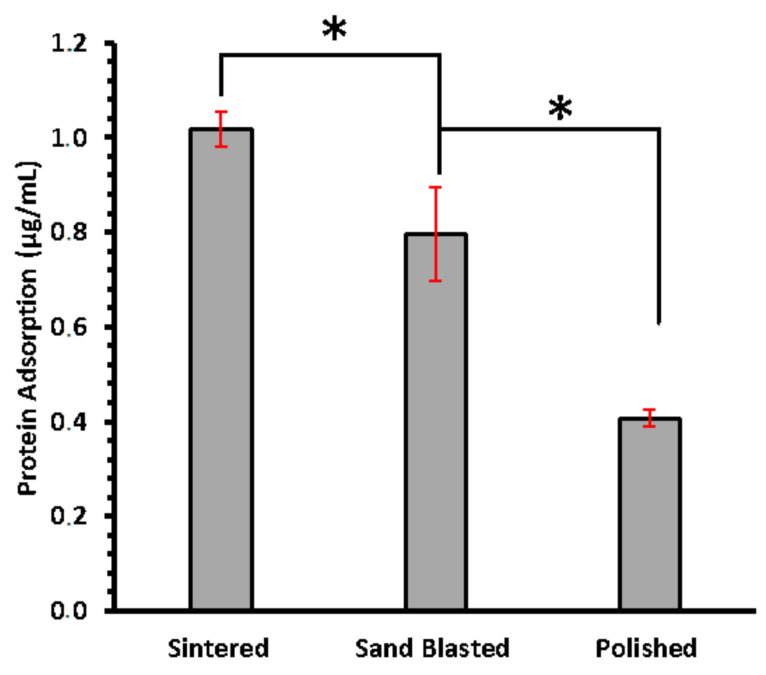
Concentration of total protein adsorbed on sintered, sand blasted, and polished surfaces of 3Y-TZP discs, measured using BCA assay. Error bars are standard deviation. Asterisk (*) sign shows significance difference.

The difference in the amount of total protein in solution before and after 24 h of incubation was measured using BCA assay. This difference is assumed as the amount adsorbed by each type of disc and is presented in Figure 3. Amount of protein adsorbed on sintered disc surfaces are the highest, averaging 1.02 µg/mL while polished surface presented the lowest total amount of protein sequestered from supernatant. Significant difference was found between sintered, and sand blasted (*p*-value of 0.05—from one tailed *t*-test). This suggested that adsorption of salivary enzyme does increase with enhancing surface roughness at nanoscale.

### 3.2. Detection of α-Amylase from Human Saliva

**Figure 4:**
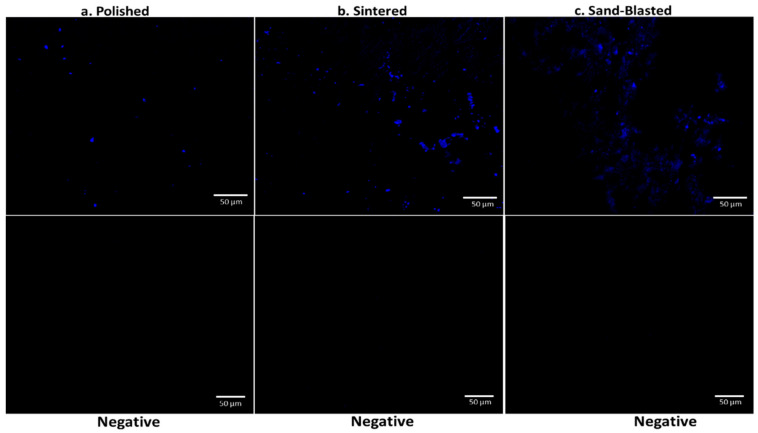
CSLM imaging of polished (**a**), sintered (**b**), and sand blasted (**c**) discs after incubation in saliva and using primary and secondary IgG for detection of α-amylase and the negative control panel for the discs.

To further investigate protein adsorption, recovered 3Y-TZP discs from saliva incubation were analyzed using immunofluorescent imaging with CSLM. Differences in the adsorption of α-amylase on discs having different surface texture is shown in CSLM imaging (Figure 4). Pronounced signal difference is found between the three variants. Polished discs of 3Y-TZP have sparsely distributed signal, whereas sintered discs with more nanoscale roughness showed an enhanced signal that is scattered over a wide area. Sand blasted discs showed more concentrated adsorption of α-amylase around the cracked regions (Figure 4c) where there is reorganization of surface structure, as confirmed from surface plots (Figure 1) [15]. 

The raw integrated density of signal analysis obtained from Figure 4 showed a similar trend as displayed in Figure 3 with highest adsorption in sintered 3Y-TZP (Figure S3). A negative control panel was performed without saliva incubation, for each type of disc. Practically, no signal was detected, with images presented in Figure 4. It confirms the specific binding of the α-amylase.

Surface features, such as nano-hillocks with edges and vertexes on zirconia, make surfaces more attractive for uptake of large biomolecules. Sintered discs of 3Y-TZP showed larger protein adsorption in BCA assay and CSLM imaging analysis than the other variants due to the presence of these surface nano features. Polished 3Y-TZP discs showed very little protein adsorption. Sand blasting with the sharp-edged alumina (Figure S4) alters surface structure by generating micron-scale roughening causing some nanoscale cracks at the edge of these micron-scale engravings (Figure 1 and Figure 2). Earlier observation on pellicle recovered with hydroxyapatite splint surfaces from humans, revealed several different morphologies of protein, IgG, and peptides from saliva at the site of adsorption. Overall thickness of the pellicle was in the range of 550–900 nm and was made up of loose fibrils, whereas globule morphology was 20–350 nm. However, small globules with diameter 9–65 nm were mainly situated next to the surface of hydroxyapatite [16]. This and our experiments support our hypothesis that, a size match exists between hard surfaces with nano-roughness below 300 nm as well as soft protein structure, including single molecules and their aggregates.

However, adsorption of salivary enzymatic molecules on the polished surface also propose another underlying mechanism together with the nano surface roughness [17], as polishing involves breakage of surface bonds when the material is removed, as a result the surface nature is altered; transforming the surface, which we initially thought as dull and monotonous attraction towards uptake of salivary biomolecules. It is recognized that proteins tend to adhere more strongly to non- polar than to polar, to high surface tension than to low surface tension, and to charged than to uncharged substrates [18,19]. Polished smoother surfaces, result in a very monotonic surface charge distribution. Hence a charge matching exists between the protein surface and the polished surface for successive adhesion of these molecules. On the other hand, presence of nano-roughening generates a variable charge distribution; together, both the factors on the sintered surface of 3Y-TZP offer better interlocking with salivary proteins with respect to their size and shape match [20]; hence, localizing protein and offering nucleating sites for further growth of supramolecular complexes leading to the formation of pellicle like tissues.

Amylase, the most abundant enzyme in human saliva, is inherently a part of glycoproteinacious developed pellicle that has the tendency to immediately adhere on the cleaned surface of implants and natural teeth. Adsorption of α-amylase is consistent with the trend of total protein adsorption on the different surfaces, with the least adsorption on polished and the highest on sintered discs. We hypothesize that the presence of surface roughness at nanoscale provides anchoring for the salivary α-amylase at one end, whereas binding directly with ligands from oral streptococcus species at the other end [21]. Survival and further colonization of these type of bacterial species then lead to biofilm formation or dental plaque. This happens primarily on the surfaces directly exposed to the salivary fluid, such as dental crown, implying the importance of surface quality. Combination of both microscale and nanoscale surface features are primarily present in sand blasted surfaces of 3Y-TZP offering intermediate level for amylase adsorption, which is in agreement with the previous studies on osteoblast differentiation [22]. However, nano roughening alone or combined with micron scale (hierarchical) below the gum level serves a different purpose in which faster healing after the implant placement and improved osseointegration is required. This study can significantly advance our understanding by filling the gap of knowledge seen in previous studies on surfaces of TZP variants. Our observations in the current study are valuable in clinical settings and can improve the handling of zirconia in the dental restoration laboratories for implant preparation and final finishing, as well as in clinical practices for healthcare providers.

## 4. Conclusions

In summary, we created three different type of surface architectures on clinically relevant 3Y-TZP, with the difference at micron and nanoscale roughness, and studied their efficacy towards initial protein adsorption from human saliva. Sintered discs of 3Y-TZP with higher surface roughness at nanoscale, possessing fine grain structure and inter grain porosity, showed enhanced total protein adsorption in comparison to sand blasted and polished surfaces. CSLM imaging of discs confirmed homogenous adsorption of salivary α-amylase on the as sintered disc. In sand blasted discs, adsorption mainly occurs at the nano-cracks. Polished discs present poor adsorption towards α-amylase with scarce fluorescence signals. This study advances our insight into the nanoscale adsorption phenomena of biologically relevant molecules on nano-roughened surfaces.

## Supplementary Materials

The following are available online at https://www.mdpi.com/article/10.3390/ma14216412/s1, Figure S1: Lay out for detection of protein on the surface of different disc surfaces of YTZ, Figure S2: Standard curves obtain in BCA assay for measuring the total protein contents of saliva at dilution used in comparing the discs, Figure S3: Confocal images presented in figure 5 were used to calculate the total fluorescence signal. In the corrected total fluorescence bar, background was measured at 8 random places in circular shape and average of these was subtracted. This background places were chosen assuming absence of any signal. Unintentional error may exist in these background measurements, an integrated density graph is also presented in parallel to corrected signal, Figure S4: Morphology and particle size of blasted media.

## References

[1] Zhang Y., Lawn B.R. (2018). Novel Zirconia Materials in Dentistry. J. Dent. Res..

[2] Cepic L.Z., Dvorak G., Piehslinger E., Georgopoulos A. (2020). In vitro adherence of Candida albicans to zirconia surfaces. Oral Dis..

[3] Rosengren Å., Pavlovic E., Oscarsson S., Krajewski A., Ravaglioli A., Piancastelli A. (2002). Plasma protein adsorption pattern on characterized ceramic biomaterials. Biomaterials.

[4] Lima E.M.C.X., Koo H., Smith A.M.V., Rosalen P.L., Cury A.A.D.B. (2008). Adsorption of salivary and serum proteins, and bacterial adherence on titanium and zirconia ceramic surfaces. Clin. Oral Implants Res..

[5] Hisbergues M., Vendeville S., Vendeville P. (2009). Review zirconia: Established facts and perspectives for a biomaterial in dental implantology. J. Biomed. Mater. Res.-Part B Appl. Biomater..

[6] Teughels W., Van Assche N., Sliepen I., Quirynen M. (2006). Effect of material characteristics and/or surface topography on biofilm development. Clin. Oral Implants Res..

[7] Moritz J., Abram A., Čekada M., Gabor U., Garvas M., Zdovc I., Dakskobler A., Cotič J., Ivičak-Kocjan K., Kocjan A. (2019). Nanoroughening of sandblasted 3Y-TZP surface by alumina coating deposition for improved osseointegration and bacteria reduction. J. Eur. Ceram. Soc..

[8] Shen J.Z., Kosmač T. (2014). Advanced Ceramics for Dentistry.

[9] Columbia B. (1996). Structure of Human Salivary a-Amylase at 1.6 A Resolution: Implications for Its Role in the Oral Cavity. Introduction.

[10] Kohavi D., Klinger A., Steinberg D., Sela M.N. (1995). Adsorption of salivary proteins onto prosthetic titanium components. J. Prosthet. Dent..

[11] Kilian M., Nyvad B. (1990). Ability to bind salivary α-amylase discriminates certain viridans group streptococcal species. J. Clin. Microbiol..

[12] Chaudhuri B., Rojek J., Vickerman M.M., Tanzer J.M., Scannapieco F.A. (2007). Interaction of salivary alpha-amylase and amylase-binding-protein A (AbpA) of Streptococcus gordonii with glucosyltransferase of S. gordonii and Streptococcus mutans. BMC Microbiol..

[13] Nikitkova A.E., Haase E.M., Scannapieco F.A. (2013). Taking the starch out of oral biofilm formation: Molecular basis and functional significance of salivary α-amylase binding to oral streptococci. Appl. Environ. Microbiol..

[14] Kiran R., Garcia F., Jimenez-pique E., Anglada M. (2013). Phase transformation and subsurface damage in 3Y-TZP after sandblasting. Dent. Mater..

[15] Gibbins H.L., Yakubov G.E., Proctor G.B., Wilson S., Carpenter G.H. (2014). What interactions drive the salivary mucosal pellicle formation?. Colloids Surf. B Biointerfaces.

[16] LIE T. (1977). Scanning and transmission electron microscope study of pellicle morphogenesis. Eur. J. Oral Sci..

[17] Celik E., Negi R.S., Bastianello M., Boll D. (2020). Tailoring the protonic conductivity of porous yttria-stabilized zirconia thin films by surface modification. Phys. Chem. Chem. Phys..

[18] Rabe M., Verdes D., Seeger S. (2011). Understanding protein adsorption phenomena at solid surfaces. Adv. Colloid Interface Sci..

[19] Limo M.J., Sola-Rabada A., Boix E., Thota V., Westcott Z.C., Puddu V., Perry C.C. (2018). Interactions between Metal Oxides and Biomolecules: From Fundamental Understanding to Applications. Chem. Rev..

[20] Kaneko T., Nagata F., Kugimiya S., Kato K. (2020). Adsorptive properties of milk proteins onto novel porous zirconia. J. Ceram. Soc. Jpn..

[21] Haase E.M., Kou Y., Sabharwal A., Liao Y.C., Lan T., Lindqvist C., Scannapieco F.A. (2017). Comparative genomics and evolution of the amylase-binding proteins of oral streptococci. BMC Microbiol..

[22] Gittens R.A., McLachlan T., Cai Y., Berner S., Tannenbaum R., Schwartz Z., Sandhage K.H., Boyan B.D. (2012). The effects of combined micron-/submicron-scale surface roughness and nanoscale features on cell proliferation and differentiation. Biomaterials.

