# Supplementary material for: Surface Effect of Nano-Roughened Yttria-Doped Zirconia on Salivary Protein Adhesion
Source: Materials (Basel). 2021 Oct 26;14(21):6412. doi: 10.3390/ma14216412 (PMC8585120; doi:10.3390/ma14216412)
Supplement: Supplementary file 1 [file materials-14-06412-s001.zip › materials-1390779-supplementary.pdf]

materials

MDPI

Communication

Surface Effect of Nano-Roughened Yttria-Doped Zirconia on Salivary Protein Adhesion

Muhammad Naeem Iqbal [1], Zhijian James Shen [1], Tore Bengtsson [2], and Mirva Eriksson [1,*]

[1] Department of Materials and Environmental Chemistry, Stockholm University, Stockholm 10691, Sweden; muhammad.iqbal@mmk.su.se (M.N.I.); zhijian.shen@mmk.su.se (Z.J.S.)
[2] Department of Molecular Biosciences, The Wenner-Gren Institute, Stockholm University, Stockholm 10691, Sweden; tore.bengtsson@su.se
* **Correspondence:** mirva.eriksson@mmk.su.se

Citation: Iqbal, M.N.; Shen, Z.J.; Bengtsson, T.; Eriksson, M. Surface Effect of Nano-Roughened Yttria-Doped Zirconia on Salivary Protein Adhesion. *Materials* **2021**, *14*, 6412. https://doi.org/10.3390/ma14216412

Academic Editors: Dinesh Agrawal

Received: 6 September 2021
Accepted: 22 October 2021
Published: 26 October 2021

Publisher's Note: MDPI stays neutral with regard to jurisdictional claims in published maps and institutional affiliations.

Copyright: © 2021 by the authors. Submitted for possible open access publication under the terms and conditions of the Creative Commons Attribution (CC BY) license (http://creativecommons.org/licenses/by/4.0/).

Microscopy Characterization:

Scanning Electron Microscopy (SEM):

SEM micrographs were taken by using a JEOL SEM 7000F(Japan). Samples were first glued on a copper double tape with observed surface on top side as opposite to the glued side. Furthermore, another single sided glued copper tape was used to connect the top of the discs with the sample holder. No Further coating was used on the disc surfaces under observation. Imaging was performed at a 5 KV accelerating voltage. SEM Images obtained were further processed through ImageJ software for the purpose of presenting a fine surface architecture shown as a 3D surface graphs in figure 1.

Confocal Microscopy:

The inverted confocal microscope Zeiss LSM 780 (Carl Zeiss AG - Germany) was utilized to image the immunolabeled detection of α-amylase from saliva on the surface of sintered, sand blasted and polished 3Y-TZP discs. Microscope was equipped with diode laser 405 nm, an Argon Laser 458/488/514 nm lines, DPSS 561 nm, and HENE 633 nm laser. The instrument is attached with a Spectral detector (GaAsp 32 channels) for acquisition of Lambda Stacks and subsequent digital separation into component dyes, three additional PMTs, with two for confocal detection. The spectral detection unit is adjustable within 390–750 nm. The images were collected using ZEN black software with Plan-Apo 20x/0.8 lens, processing of data was done in ZEN blue software. Results of which are displayed in figure 4.

Roughness Parameter Characterization:

Surface topographic measurement was performed using optical profiler equipped with interferometry (White-light interferometer (WLI), Gesellschaft fur Bild-und Signal-Verarbeitung (GBS) mbH – Germany), for all sintered, sand blasted and polished surfaces. It measures 3D surface architecture in a non-contact fashion and aided with the software packages for data processing. SmartVIS3D software was used for Data collection with provided Nikon Mirau lens. Smallest accessible increment of scanning for the step size was used for acquisition in z-direction. Measuring field with laser on the surface of the discs was around 1.7 mm × 1.4 mm. All the images after stitching were used to perform the roughness parameter and topographic measurements of the discs in Mountain Map (an Imaging topography software) provided by Digital Surf sarl (France). After correcting the x, y tilts, data was acquired, for all the data processing 0.25 mm Gaussian pass filter was applied to separate the roughness from waviness. Extracted roughness values are presented in the figure 2.

BCA Assay:

Analysis for total protein contents from the supernatant was performed with BCA Assay kit (Quanti Pro™–Sigma-Aldrich). Spectrophotometer measurement was performed at a 562 nm wavelength. All the measurements were performed on EnSpire™ from PerkinElmer in a 96 well plate.

Bicinchoninic acid (BCA) protein assay was used to determine the total protein concentration in a way to estimate the amount proteins from human saliva adsorbed on the three different surfaces[i]. Protein concentration was determined using a reactive solution of BCA and $CuSO_4$ of green coloration, provided with in the assay kit. Cu^{2+} ions in $CuSO_4$ are quantitively reduced to Cu^{+} by the proteins in the cell suspension. Thereby reduced Cu^{+} ion forms a complex with BCA, hence cause a colour change. The coloration of this complex is directly proportional to the protein contents. A standard protein concentration curve was developed using bovine serum albumin (BSA) as a standard. Furthermore, native human salivary alpha-amylase purchased from abcam (product ID ab77875) was used also a standard for estimation of amount of proteins in human saliva. Both proteins were serial diluted with known amounts for running in assay. In parallel freshly collected saliva was serial diluted and tested for total protein concentration against these dilutions. Prior to dilution, freshly collected saliva from volunteers was spin down at 5000 rpm (4500 rcf) for 5 mins to remove any food debris. Both the proteins BSA and human salivary alpha-amylase were also processed at the same speed in parallel when taken out from 4 °C storage. 1 L PBS buffer of pH 6.4 was prepared from commercially bought tablets and used in all dilution and processing. Finally, a stock of 1/46 dilution (1part saliva with 46 part PBS buffer) was chosen from these dilutions and prepared for incubating all the discs. 3Y-TZP Discs were placed in a 48 well cell culture plate with wells labelled for sintered, sand blasted, polished or saliva. Each well was filled with 2.5 mL of diluted saliva and incubated in 37 °C preheated oven for about 24 hours. At time zero, a sample of 60 µL(3x) sample was collected and placed in a 95 well microplate and placed at −20 °C until 24 hour sample collection. Carefully drawn supernatant solution from each sintered, sand blasted, polished and saliva wells on to the same micro plate with time zero sample, collection volume was 60 µL (3x). Freshly, prepared BCA mixture from the Kit (5 mL A, 5 mL B and 0.2 mL copper solution) and vortexed for 30 seconds. Only buffer solution with BCA was chosen as background measurement of absorbance. Plate was placed in 60 C preheated oven for 2 hours. To avoid any volume loss, micro plate was sealed with Biorad Microseal® film and secured with the cover. After taking out from the oven plate was cooled to room temperature, centrifuged to draw all the solution to bottom and carefully removed the seal. Plate was loaded in the spectrophotometer, prior to taking the absorbance, shake time and shake speed of 5 second and 60 rpm respectively was set. The protein concentration was determined from the absorbance at 562 m. Average of absorbance without any sample that is buffer was used for background correction and subtracted from all the measurements. A difference between absorbance at time zero and 24 hour was assumed to be directly related with the amount of protein taken up on each type of discs of 3Y-TZP surface. Slope from the human salivary alpha-amylase standard curve was used to calculate the amount of protein in saliva at each serial dilution that is presented in the supplementary information.

Immunolabelling:

Immunolabelling technique was used to detect the adsorbed protein from human saliva on the surface of sintered, sand blasted and polished discs. In this technique, two antibodies were used for the detection of alpha-amylase from human saliva; primary antibody of Rabbit anti-human salivary alpha-amylase (product ID ab125230) and secondary antibody were donkey anti-rabbit tagged with Alexafluor-405 (product ID ab175649). Freshly collected human saliva was c processed in similar fashion as in BCA assay and supernatant was further diluted to 43x with pH 6.4 buffer. This stock was used for incubation of sintered, sand blasted and polished 3Y-TPZ discs. Initially, incubation was at room temperature for 3–4 hours followed by overnight at 4 °C in total of 24 hours. 48 well

cell culture plate was used incubation and without any shaking or agitation. 2.5 mL saliva solution was poured in the tubes containing sintered, sand blasted or polished discs. After 24 hours of incubation time, solution was removed using micropipette and fresh 2.5 mL buffer solution of same pH was poured for a quick rinse. In the second pour of buffer, discs were kept at shaking table for maximum of 5 minutes in the same well plate. Collected 3Y-TZP discs were than immersed in primary antibody with 1/100x dilution for 3–4 hours, following the same rinsing and shaking steps. Lastly, incubation in Alexa fluor 405 tagged secondary antibody was completed after overnight incubation with same rinsing and washing step at the end. Here figure 4 presents a general layout of the immunolabelling procedure. A negative control panel was also with 3Y-TZP discs of all three variants processed in parallel in spate well without saliva, firstly immersed in primary antibody for 24 hours (3 hours at room temperature and rest at 4 °C). Similar procedure was repeated with the secondary antibody at same dilution level for same time. After final rinsing and washing step, discs were processed for CSLM imaging.

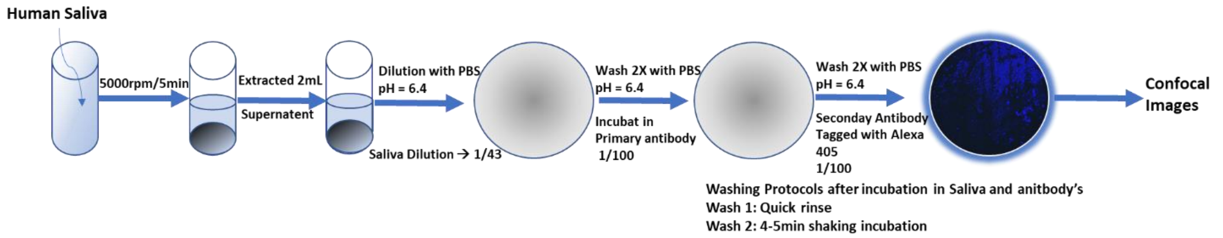

Figure S1. Lay out for detection of protein on the surface of different disc surfaces of YTZ.

BCA Assay Measurement

BCA Measurements for total protein contents in saliva. BSA and human salivary alpha-amylase is used as standard proteins.

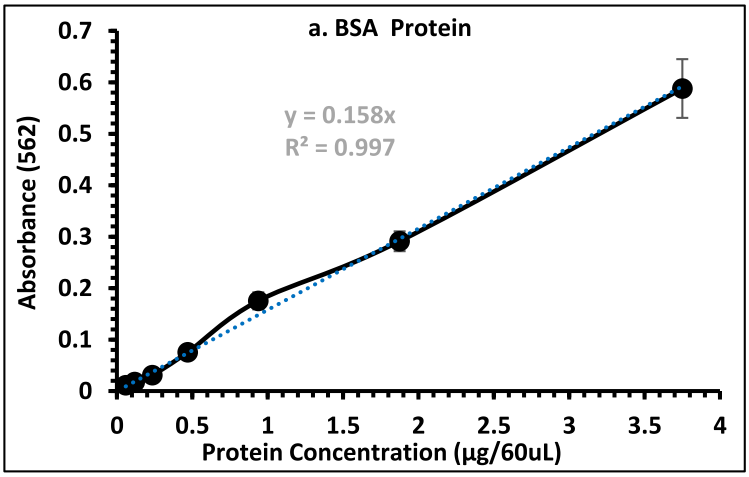

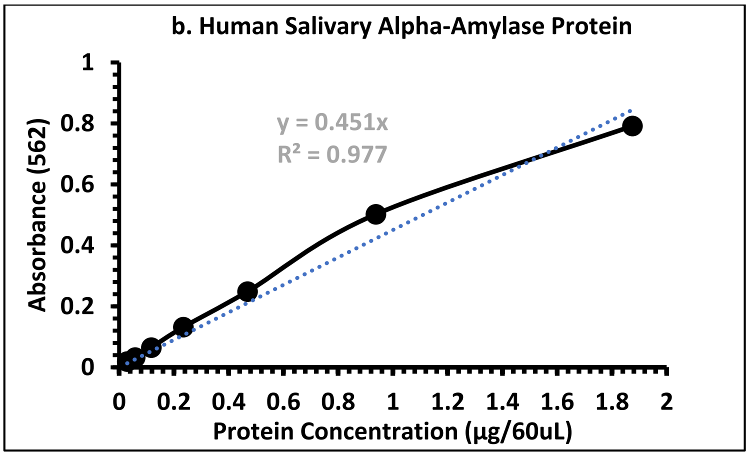

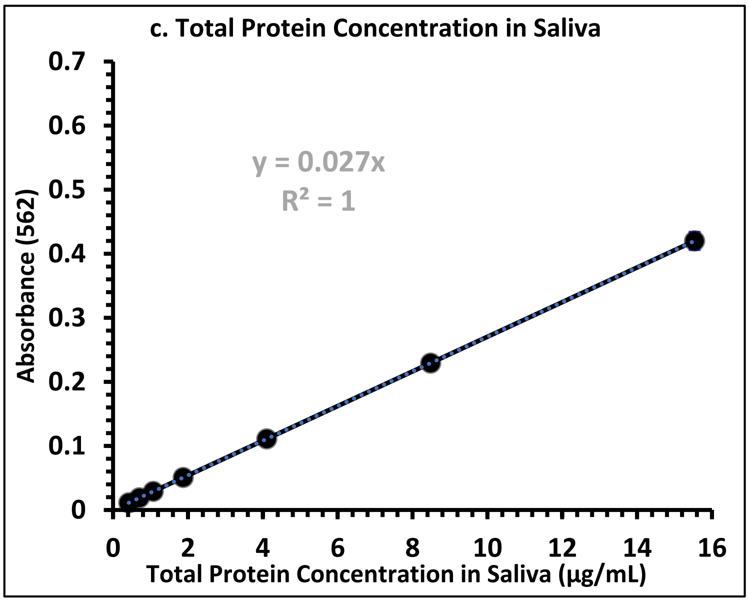

Figure S2. Standard curves obtain in BCA assay for measuring the total protein contents of saliva at dilution used in comparing the discs.

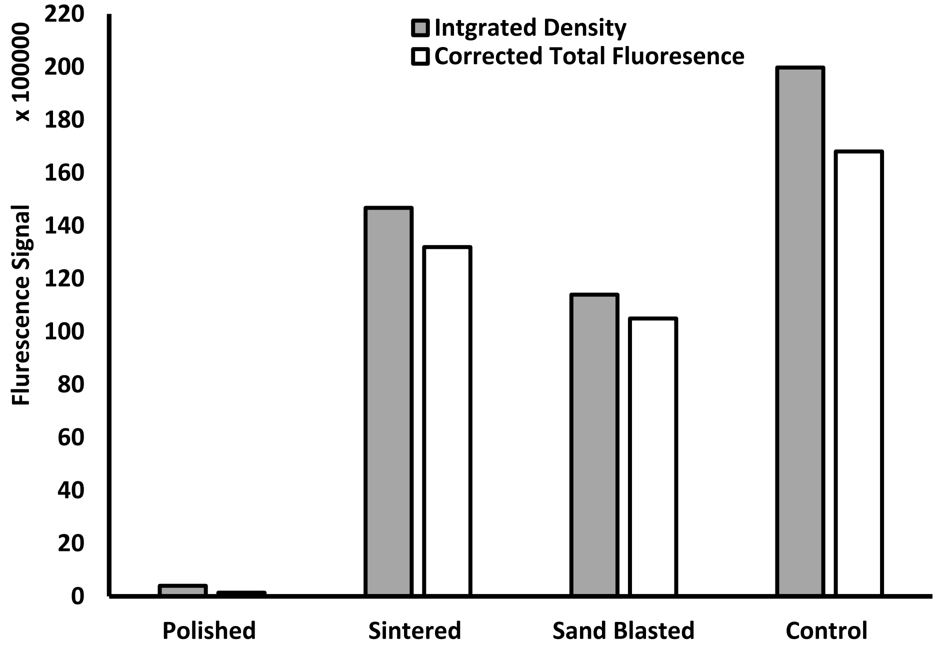

Figure S3. Confocal images presented in figure 5 were used to calculate the total fluorescence signal. In the corrected total fluorescence bar, background was measured at 8 random places in circular shape and average of these was subtracted. This background places were chosen assuming absence of any signal. Unintentional error may exist in these background measurements, an integrated density graph is also presented in parallel to corrected signal.

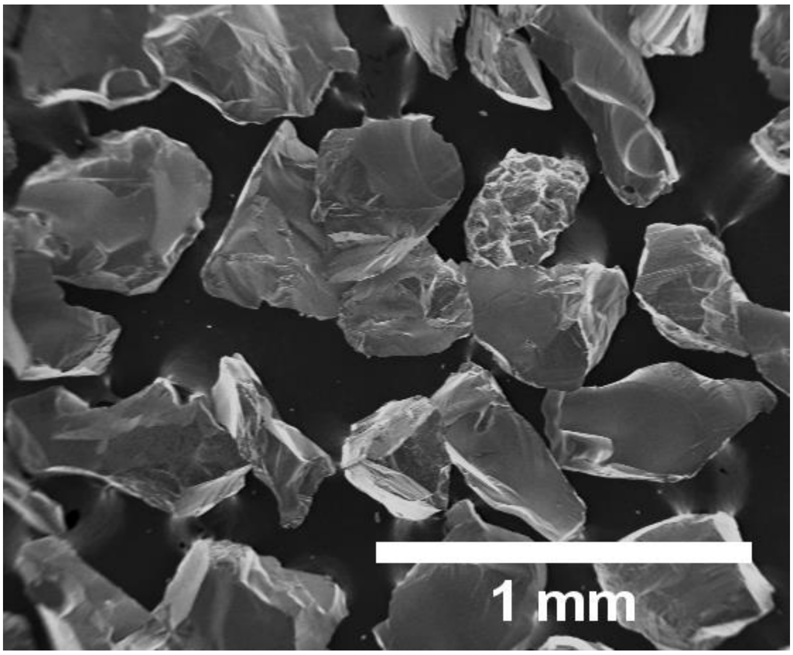

Figure S4. Morphology and particle size of blasted media.

---

[i] P.K. Smith, R.I. Krohn, G.T. Hermanson, A.K. Mallia, F.H. Gartner, M.D. Provenzano, E.K. Fujimoto, N.M. Goeke, B.J. Olson, D.C. Klenk; Measurement of Protein Using Bicinchoninic Acid; Anal. Biochem, 150 (1985):76-85.
